# Cyclin D1 G870A Polymorphism Contributes to Colorectal Cancer Susceptibility: Evidence from a Systematic Review of 22 Case-Control Studies

**DOI:** 10.1371/journal.pone.0036813

**Published:** 2012-05-11

**Authors:** Yongzhi Yang, Feng Wang, Chenzhang Shi, Yang Zou, Huanlong Qin, Yanlei Ma

**Affiliations:** 1 Department of Surgery, Shanghai Tenth People’s Hospital affiliated with Tongji University, Shanghai, People’s Republic of China; 2 Department of Surgery, The Sixth People’s Hospital affiliated with Shanghai Jiao Tong University, Shanghai, People’s Republic of China; Ohio State University Medical Center, United States of America

## Abstract

**Background:**

Cyclin D1 (*CCND1*) plays a vital role in cancer cell cycle progression. Numerous epidemiological studies have evaluated the association between the *CCND1* G870A polymorphism and the risk of colorectal cancer. However, these studies have yielded conflicting results. To derive a more precise estimation of this association, we conducted a meta-analysis and systematic review.

**Methodology/Principal Findings:**

A comprehensive search was conducted to identify eligible studies of the *CCND1* G870A polymorphism and colorectal cancer risk. Pooled odds ratios (ORs) with 95% confidence intervals (CIs) were derived from a fixed effect or random effect model. We applied a grading system (Venice criteria) that assessed the epidemiological strength of the association. A total of 22 publications that included 6157 cases and 8198 controls were identified. We found that the *CCND1* G870A polymorphism was significantly associated with overall colorectal cancer risk (homozygote genetic model: OR = 1.130, 95% CI = 1.023–1.248, P = 0.016; heterozygote genetic model: OR = 1.124, 95% CI = 1.030–1.226, P = 0.009; dominant genetic model: OR = 1.127, 95% CI = 1.037–1.224, P = 0.005). After further stratified analyses, the increased risk was observed only in the subgroups of hospital-based studies, PCR-RFLP genotyping methods, sporadic colorectal cancer, and Caucasian ethnicity.

**Conclusions:**

The available evidence demonstrates that the *CCND1* 870A allele might be a low-penetrant risk factor for colorectal cancer.

## Introduction

Colorectal cancer (CRC) is the second most common type of cancer in women and the third most common type in men in the United States and Europe [1], [2]. The multistep carcinogenesis of the adenoma-carcinoma sequence is determined by caretaker molecular pathways, and this conventional theory is also thought to describe colorectal oncogenesis [3], [4]. However, it is now commonly accepted that the pathogenesis of CRC involves the multi-factorial interactions of environmental triggers and genetic susceptibility [5]. A recent study have revealed that approximately 35% of CRC cases can be attributed to inherited genetic susceptibility [5].

The adenine-to-guanine (A/G) substitution at nucleotide 870 (*CCND1* G870A polymorphism, rs603965) and excessive cyclin D1 activity are common in numerous human tumors, including breast cancer, lung cancer, head and neck cancers, gastric cancer, gynecological cancers, blood-related cancers, and CRC [6], [7]. Although various studies have linked the *CCND1* G870A polymorphism to increased CRC risk, the results remain controversial. To further investigate the combined effect of the *CCND1* G870A polymorphism on CRC susceptibility, we performed a meta-analysis and systematic review.

## Methods

### Identification and Eligibility of Relevant Studies

All published literature investigating an association between the *CCND1* G870A polymorphism and colorectal cancer risk were eligible. We searched for studies using the PubMed database up to October 2011. The relevant search terms “G870A”, “A870G”, “*CCND1*”, “cyclin D1”, “polymorphism”, ”cancer”, “colorectal”, “colonic”, “colon”, “rectal”, “rectum”, and “humans” were used. Both free text and a MeSH search for keywords were employed. We also manually searched the reference lists in selected articles and the abstracts published at major international conferences. Abstracts that were not written in English were excluded. All the studies met the following criteria: (1) the *CCND1* G870A polymorphism was determined; (2) the outcome had to be colorectal cancer in humans. The major exclusion criteria were (1) reviews, tutorials, letters, and editorials; (2) duplicate data; (3) not a case-control design; (4) insufficient data were reported as cyclin D1 expression levels were provided without genotype data; (5) overlapping data and data superseded by the latest reports.

### Data Extraction

Data were extracted independently and crosschecked against the research consensus. The following variables were recorded: the first author’s last name; publication year; region/country where the study was performed; participant gender; ethnicity (included Caucasian, Asian and Mixed) of the study population; epidemiological type of colorectal cancer (included hereditary nonpolyposis colorectal cancer (HNPCC), sporadic colorectal cancer (sCRC), and sporadic colonic cancer (sCC)); histopathological subgroup information if known (included Dukes’ stage (A/B and C/D) and degree of differentiation (well/moderate, moderate and poor)); control source (family-based study (FB), population-based study (PB), and hospital-based study (HB)); genotyping method (polymerase chain reaction (PCR) single-stranded conformation polymorphism (PCR-SSCP), PCR restriction fragment length polymorphism (PCR-RFLP), high-performance liquid chromatography (HPLC), TaqMan PCR, and DNA sequencing); sample size (total cases and controls as well as the numbers of cases and controls with G/G, G/A, and A/A genotypes); and the P value of the Hardy-Weinberg equilibrium in the control group. Only the latest studies were included when the data sets overlapped or were duplicated. The primary authors were contacted to provide additional information when necessary. Study identification and data extraction were conducted independently by three investigators and checked for accuracy by one author.

### Statistical Analysis

Dichotomous variables were pooled using an odds ratio (OR). The summary OR was replaced by the risk difference (RD) if one of the studies reported no events in either the case group or the control group.

The wild type G/G genotype was considered as a reference. Pooled effects were calculated for a homozygote comparison model (A/A vs. G/G), a heterozygote comparison model (G/A vs. G/G), a dominant model (G/A+A/A vs. G/G), and a recessive model (A/A vs. G/G+G/A).

The statistical heterogeneity between included studies was determined using the chi-square-based Q-test [8], [9]. According to the Higgins’ I^2^ statistic, heterogeneity was defined as low or moderate if less than 50% and high if greater than 50% [8]. A fixed effect model was applied using the Mantel-Haenszel method for low or moderate statistical heterogeneous studies [10]. A random effect model, which assumed that the studies involved came from a random sample of a hypothetical population of studies that took into account heterogeneity, was used when heterogeneity was high [11]. A Galbraith plot was created to graphically assess the extent of heterogeneity between studies from the current meta-analysis [12], [13]. A L’Abbé plot was used for the additionally assessment of colorectal cancer risk [14], [15]. The Hardy-Weinberg equilibrium (HWE) was determined using the chi-square test in the control groups [16].

Sensitivity analyses were conducted either by replacing a value of effect with another or removing individual studies from the data set. Sensitivity analyses were also performed by excluding studies in which the genotype frequencies in the controls significantly deviated from the HWE. We conducted subgroup analyses of the study design, cancer type, cancer location, ethnicity, Dukes’ stage, degree of differentiation, gender and genotyping method to investigate potential sources of heterogeneity.

Publication bias among the included studies was assessed graphically using a Begg’s funnel plot [17]. Additionally, publication bias was also evaluated statistically with an Egger’s test [18].

The study confidence interval (CI) was established at 95%. Two-tailed P values of less than 0.05 were considered statistically significant. All statistical analyses were performed using the STATA version 11.0 software (Stata Corporation, College Station, TX).

### Assessment of Cumulative Evidence

The Venice criteria [19] were developed by the Human Genome Epidemiology Network (HuGENet) Working Group to assess the cumulative epidemiological strength of genetic association studies; these same criteria were applied in this study. Following the Venice criteria, our meta-analysis was graded based on three categories: (1) the amount of evidence (sample sizes of cases and controls that were greater than 1000, 100–1000, or less than 100 were assigned a grade of A, B, or C, respectively); (2) the extent of replication (a Higgins’ I^2^ statistic [8] that was less than 25%, 25% – 50% or greater than 50% was assigned a grade of A, B, or C, respectively); (3) protection from bias (a grade of A was assigned if there was no observable bias, a grade of B was assigned if bias could be present or could explain the presence of the association; a grade of C was assigned if bias was considerable and had an effect even the presence or absence of the association).

## Results

### Characteristics of the Studies

**Figure 1:**
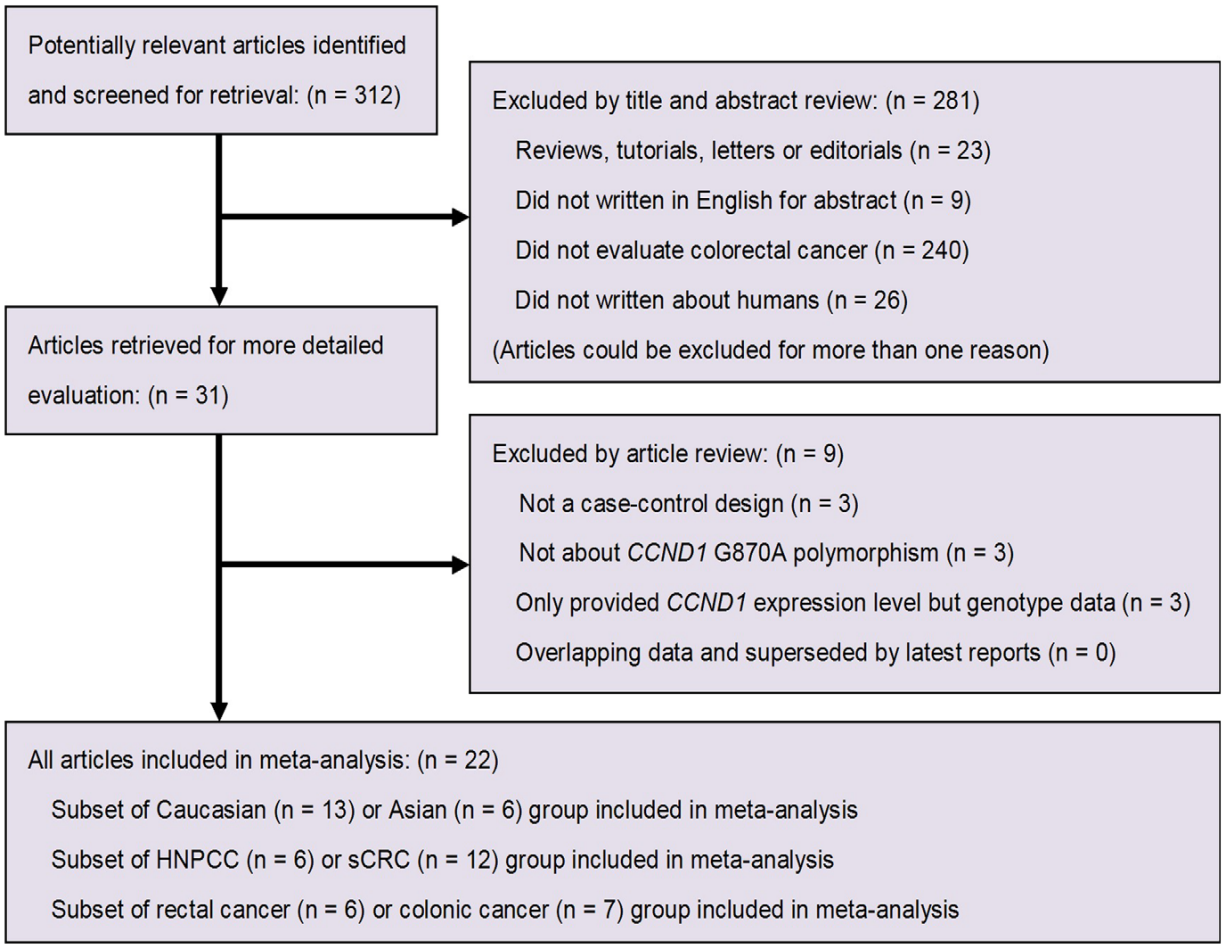
Flow chart of study selection according to MOOSE guidelines [**42**].

Through literature search and selection, a total of 22 publications [20], [21], [22], [23], [24], [25], [26], [27], [28], [29], [30], [31], [32], [33], [34], [35], [36], [37], [38], [39], [40], [41] including 6157 cases and 8198 controls comparing the *CCND1* G870A polymorphism and colorectal cancer susceptibility were identified based on MOOSE (Meta-analysis Of Observational Studies in Epidemiology) guidelines [42]. Two studies [24], [35] investigated both HNPCC and sCRC, and the genotype frequencies were therefore separated into three types: Mixed, HNPCC, and sCRC. One article [26] mentioned two independent populations (Asians and Caucasians), and the study was thus treated as three separate estimates: Mixed, Asians, and Caucasians. A flow chart of the inclusion and exclusion criteria is presented in **Figure 1**.

Five articles [20], [26], [34], [37], [39] showed mixed or missing ethnicity data. Nine studies [24], [30], [32], [33], [34], [35], [37], [40], [41] showed mixed types of cancer data. Of the 22 included studies, 2 were family-based [20], [22], 11 were population-based [21], [23], [24], [26], [28], [31], [32], [33], [37], [38], [40], and 9 were hospital-based [25], [27], [29], [30], [34], [35], [36], [39], [41]. Multiple genotyping methods were employed in the studies and included PCR-RFLP, PCR-SSCP, HLC, TaqMan PCR, and DNA sequencing. The distribution of genotypes in the controls of all studies was consistent with Hardy-Weinberg equilibrium except in one study [29]. Characteristics of the studies included are summarized in **Table 1**.

**Table 1:** Characteristics of the studies included in the meta-analysis.

First author (Year)	Country	Ethnicity	Type of cancer	Source of controls	Genotyping method	Total, N		GG genotype, N		GA genotype, N		AA genotype, N		HWE	Reference
						Cases	Controls	Cases	Controls	Cases	Controls	Cases	Controls		
Kong (2000)	US	Mixed	HNPCC	FB	PCR-SSCP	49	37	9	10	36	21	4	6	0.51	[20]
McKay (2000)	UK	Caucasian	sCRC	PB	PCR-RFLP	100	101	25	34	58	50	17	17	0.849	[21]
Bala (2001)	Finland	Caucasian	HNPCC	FB	PCR-SSCP	146	186	50	47	70	97	26	42	0.551	[22]
Kong (2001)	US	Caucasian	sCRC	PB	PCR-SSCP	156	152	36	45	71	84	49	23	0.112	[20]
Porter (2002)	UK	Caucasian	Mixed	PB	PCR-RFLP	334	171	85	60	175	81	74	30	0.768	[24]
Porter (2002)	UK	Caucasian	HNPCC	PB	PCR-RFLP	99	171	30	60	47	81	22	30	0.768	[24]
Porter (2002)	UK	Caucasian	sCRC	PB	PCR-RFLP	128	171	34	60	65	81	29	30	0.768	[24]
Grieu (2003)	Australia	Caucasian	sCRC	HB	PCR-SSCP	569	327	142	90	313	158	114	79	0.556	[25]
Le Marchand (2003)	US	Mixed	Mixed	PB	PCR-RFLP	504	624	109	164	253	315	142	145	0.792	[26]
Le Marchand (2003)	US	Caucasian	sCRC	PB	PCR-RFLP	138	161	29	50	75	85	34	26	0.311	[26]
Le Marchand (2003)	US	Asian	sCRC	PB	PCR-RFLP	296	380	75	96	143	195	78	89	0.603	[26]
Lewis (2003)	US	Caucasian	sCRC	HB	PCR-RFLP	161	213	51	84	84	98	26	31	0.781	[27]
Hong (2005)	Singapore	Asian	sCRC	PB	PCR-RFLP	254	101	55	12	128	50	71	39	0.505	[28]
Huang (2006)	Taiwan	Asian	sCRC	HB	PCR-RFLP	831	1052	126	199	411	464	294	389	0.004	[29]
Jiang (2006)	India	Asian	Mixed	HB	PCR-RFLP	301	291	46	56	130	145	125	90	0.86	[30]
Kruger (2006)	Germany	Caucasian	HNPCC	PB	Multiplex PCR	315	245	110	73	144	121	61	51	0.947	[31]
Probst-Hensch (2006)	Singapore	Asian	Mixed	PB	TaqMan PCR	300	1169	56	207	132	548	112	414	0.272	[32]
Schernhammer (2006)	US	Caucasian	Mixed	PB	TaqMan PCR	610	1237	125	264	311	593	174	380	0.25	[33]
Forones (2008)	Brazil	Mixed	Mixed	HB	PCR-RFLP	123	120	36	34	66	67	21	19	0.141	[34]
Grunhage (2008)	Germany	Caucasian	Mixed	HB	PCR-RFLP	194	218	37	48	93	109	64	61	0.958	[35]
Grunhage (2008)	Germany	Caucasian	HNPCC	HB	PCR-RFLP	98	218	13	48	50	109	35	61	0.958	[35]
Grunhage (2008)	Germany	Caucasian	sCRC	HB	PCR-RFLP	96	218	24	48	43	109	29	61	0.958	[35]
Talseth (2008)	Australia/Poland	Caucasian	HNPCC	HB	TaqMan PCR	157	153	34	42	78	80	45	31	0.527	[36]
Tan (2008)	Germany	Mixed	Mixed	PB	PCR-RFLP	498	600	120	147	263	310	115	143	0.414	[37]
Jelonek (2010)	Poland	Caucasian	sCC	PB	PCR-RFLP	50	153	12	44	33	71	5	38	0.383	[38]
Kanaan (2010)	US	NS	sCRC	HB	PCR-HLC	75	93	19	24	39	48	17	21	0.748	[39]
Liu (2010)	China	Asian	Mixed	PB	PCR-RFLP	373	838	66	160	187	429	120	249	0.303	[40]
Yaylim-Eraltan (2010)	Turkey	Caucasian	Mixed	HB	PCR-RFLP	57	117	9	29	28	60	20	28	0.781	[41]

HWE: Hardy–Weinberg equilibrium; US: United States; UK: United Kingdom; HNPCC: hereditary nonpolyposis colorectal cancer; sCRC: sporadic colorectal cancer; sCC: sporadic colonic cancer; FB: family-based study; PB: population-based study; HB: hospital-based study; PCR: polymerase chain reaction; SSCP: single-stranded conformation polymorphism; RFLP: restriction fragment length polymorphism; HPLC: high-performance liquid chromatography.

### Heterogeneity Analysis

The genotype data in the 22 studies were homogenous for the heterozygote genetic model (G/A vs. G/G: Q-test = 23.65, P = 0.310, I^2^ = 11.20) and the dominant genetic model (G/A+A/A vs. G/G: Q-test = 27.93, P = 0.142, I^2^ = 24.80), but heterogeneity was significant for the homozygote genetic model (A/A vs. G/G: Q-test = 39.53, P = 0.008, I^2^ = 46.90) and the recessive genetic model (A/A vs. G/G+G/A: Q-test = 27.93, P = 0.142, I^2^ = 52.70).

**Figure 2:**
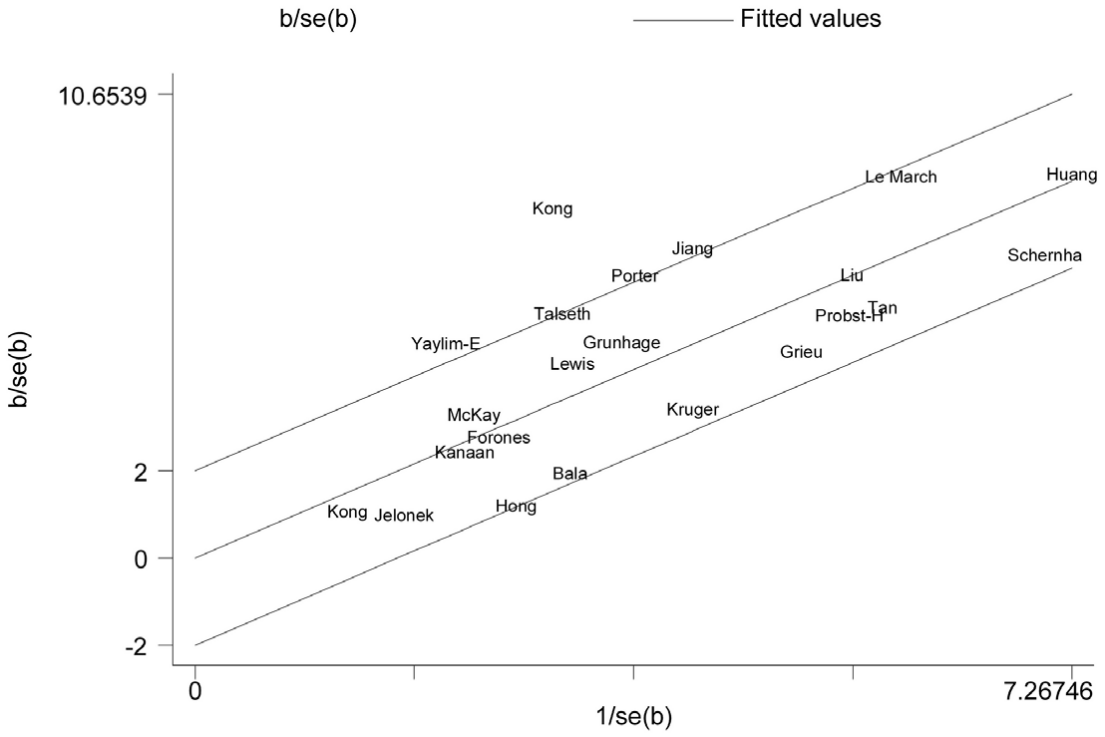
Galbraith plot [**12**] analysis of the amount of heterogeneity from all the included studies (AA vs. GG). The y-axis shows the ratio of the log OR to its standard error (SE), and the x-axis shows the reciprocal of the SE. Each study is represented by the name of the first author. A regression line runs centrally through the name. At a 2 standard deviation distance parallel to the regression line, the 2 lines create an interval. Studies lacking in heterogeneity would lie within the 95% confidence interval (positioned 2 units above and below the central regression line).

Galbraith plot analyses of all included studies were used to assess the potential sources of heterogeneity. Two studies [20], [41] were found to be contributors of heterogeneity in the homozygote comparison model (**Figure 2**).

### Association of the *CCND1* G870A Polymorphism with CRC Susceptibility

The multivariable-adjusted ORs for each study and the OR for the combination of all the studies are shown in **Table 2**; these ORs were used to determine the association of the G870A polymorphism with CRC susceptibility. A significant association of the G870A polymorphism with CRC susceptibility was observed in the homozygote comparison model, the heterozygote comparison model, and the dominant model when all the studies were considered (A/A vs. G/G: OR = 1.130, 95% CI = 1.023–1.248, P = 0.016; G/A vs. G/G: OR = 1.124, 95% CI = 1.030–1.226, P = 0.009; G/A+A/A vs. G/G: OR = 1.127, 95% CI = 1.037–1.224, P = 0.005), However, the association was not observed in the recessive genetic model (A/A vs. G/G+G/A: OR = 1.067, 95% CI = 0.941–1.210, P = 0.311).

**Table 2:** Meta-analysis of the association between the *CCND1* G870A polymorphism and colorectal cancer risk.

Study group	Homozygote comparison: A/A vs. G/G				Heterozygote comparison: G/A vs. G/G				Dominant model: G/A+A/A vs. G/G				Recessive model: A/A vs. G/A+G/G				P_E_ >0.05
	OR (95% CI)	P	Chi_H_^2^ (P_H_)	I^2^, %	OR (95% CI)	P	Chi_H_^2^ (P_H_)	I^2^, %	OR (95% CI)	P	Chi_H_^2^ (P_H_)	I^2^, %	OR (95% CI)	P	Chi_H_^2^ (P_H_)	I^2^, %	
Total	1.130(1.023 – 1.248)	0.016	39.53(0.008)	46.90	1.124 (1.030 – 1.226)	0.009	23.65 (0.310)	11.20	1.127(1.037 – 1.224)	0.005	27.93 (0.142)	24.80	1.067(0.941 – 1.210)	0.311	44.42 (0.002)	52.70	Y
Study design: PB	1.092(0.872 – 1.367)	0.442	25.62(0.004)	61.00	1.073 (0.959 – 1.201)	0.22	13.08 (0.220)	23.50	1.082(0.973 – 1.204)	0.147	16.73 (0.081)	40.20	1.044(0.944 – 1.154)	0.405	26.05 (0.004)	61.60	Y
Study design: HB	1.260(1.072 – 1.482)	0.005	10.06(0.345)	10.60	1.249 (1.082 – 1.442)	0.002	5.76 (0.764)	0.00	1.252(1.093 – 1.433)	0.001	5.94 (0.746)	0.00	1.079(0.955 – 1.219)	0.224	15.90 (0.069)	43.40	Y
Type of cancer: HNPCC	1.132(0.728 – 1.761)	0.581	11.83(0.037)	57.70	0.984 (0.790 – 1.227)	0.886	8.06 (0.153)	38.00	1.085(0.779 – 1.510)	0.63	11.03 (0.051)	54.70	1.096(0.877 – 1.371)	0.42	7.64 (0.177)	34.50	Y
Type of cancer: sCRC	1.160(0.889 – 1.514)	0.273	23.65(0.009)	57.70	1.204 (1.053 – 1.376)	0.007	12.24 (0.269)	18.30	1.188(1.046 – 1.348)	0.008	13.45 (0.200)	25.60	1.058(0.841 – 1.333)	0.629	27.61 (0.002)	63.80	Y
Location: Colon	1.228(0.963 – 1.567)	0.098	4.88(0.430)	0.00	0.984 (0.661 – 1.465)	0.938	13.69 (0.018)	63.50	1.112(0.947 – 1.304)	0.194	10.81 (0.147)	35.20	1.219(0.880 – 1.689)	0.234	10.74 (0.057)	53.50	Y
Location: Rectum	1.177(0.645 – 2.149)	0.595	15.07(0.005)	73.50	0.836 (0.385 – 1.814)	0.65	32.81 (<0.001)	87.80	0.913(0.500 – 1.664)	0.766	39.89 (<0.001)	87.50	1.224(1.001 – 1.497)	0.048	6.37 (0.173)	37.20	Y
Ethnicity: Asian	1.093(0.854 – 1.399)	0.48	11.14(0.049)	55.10	1.073 (0.927 – 1.243)	0.344	8.90 (0.113)	43.80	1.09(0.949 – 1.251)	0.223	9.42 (0.093)	46.90	1.068(0.883 – 1.292)	0.498	12.49 (0.029)	60.00	Y
Ethnicity: Caucasian (all)	1.306(0.996 – 1.713)	0.053	29.79(0.003)	59.70	1.145 (1.004 – 1.306)	0.043	16.63 (0.164)	27.90	1.162(1.026 – 1.316)	0.018	21.58 (0.043)	44.40	1.181(0.951 – 1.465)	0.132	27.9 (0.006)	57.00	Y
Ethnicity: Caucasian (HNPCC)	1.170(0.725 – 1.888)	0.521	11.59(0.021)	65.50	0.954 (0.762 – 1.196)	0.685	6.45 (0.168)	38.00	1.049(0.737 – 1.492)	0.791	10.09 (0.039)	60.40	1.125(0.897 – 1.411)	0.31	5.97 (0.201)	33.00	Y
Ethnicity: Caucasian (sCRC)	1.511(1.158 – 1.972)	0.002	10.21(0.116)	41.20	1.307 (1.057 – 1.617)	0.014	4.57 (0.600)	0.00	1.369(1.118 – 1.676)	0.002	3.71 (0.716)	0.00	1.249(0.865 – 1.805)	0.236	14.72 (0.023)	59.20	Y
Dukes’ stage: A/B	1.114(0.895 – 1.385)	0.334	1.76(0.623)	0.00	1.072 (0.876 – 1.312)	0.498	5.04 (0.169)	40.50	1.061(0.883 – 1.275)	0.529	4.41 (0.353)	9.30	1.052(0.893 – 1.241)	0.544	4.02 (0.259)	25.40	Y
Dukes’ stage: C/D	1.275(1.007 – 1.613)	0.043	5.23(0.156)	42.60	1.365 (1.097 – 1.698)	0.005	1.07 (0.785)	0.00%	1.105(0.754 – 1.618)	0.609	12.17 (0.016)	67.10	1.020(0.861 – 1.209)	0.816	5.60 (0.133)	46.40	Y
Degree of differentiation:Well/Moderate	1.199(0.932 – 1.541)	0.157	0.01(0.996)	0.00	1.337 (1.063 – 1.682)	0.013	1.38 (0.501)	0.00	1.022(0.679 – 1.538)	0.916	7.44 (0.059)	59.70	0.948(0.791 – 1.137)	0.556	0.81 (0.666)	0.00	Y
Degree of differentiation:Poor	*0.079(−0.369 – 0.527)	0.73	16.75(<0.001)	88.10	*0.072 (−0.298 – 0.443)	0.702	18.67 (<0.001)	89.30	*0.004(−0.228 – 0.236)	0.972	26.19 (<0.001)	88.50	*−0.004(−0.111 – 0.104)	0.943	3.56 (0.168)	43.80	Y
Gender: Female	1.141(0.835 – 1.559)	0.408	2.10(0.552)	0.00	1.290 (0.975 – 1.708)	0.074	2.84 (0.417)	0.00	1.282(1.003 – 1.639)	0.047	3.99 (0.407)	0.00	0.932(0.743 – 1.170)	0.545	0.48 (0.924)	0.00	Y
Gender: Male	1.318(0.991 – 1.752)	0.058	5.85(0.119)	48.70	1.393 (1.073 – 1.809)	0.013	2.90 (0.407)	0.00	1.359(1.080 – 1.710)	0.009	4.02 (0.403)	0.50	1.237(0.770 – 1.986)	0.379	6.94 (0.074)	56.80	Y
Genotyping method: PCR-RFLP	1.262 (1.126 – 1.415)	<0.001	26.80(0.083)	32.8	1.190 (1.076 – 1.315)	0.001	17.40 (0.496)	0.00	1.216(1.106 – 1.337)	<0.001	20.29 (0.317)	11.3	1.118(1.023 – 1.221)	0.014	27.62 (0.068)	34.8	Y
Genotyping method: PCR-SSCP	1.050 (0.539 – 2.047)	0.886	11.59(0.009)	74.1	1.080 (0.852 – 1.369)	0.527	5.23 (0.156)	42.7	1.070(0.745 – 1.538)	0.713	6.17 (0.104)	51.4	0.992(0.516 – 1.907)	0.980	15.41 (0.001)	80.5	Y
Genotyping method: TaqMan PCR	1.044 (0.848 – 1.286)	0.684	3.06(0.216)	34.7	1.049 (0.865 – 1.272)	0.626	1.26 (0.533)	0.00	1.049(0.875 – 1.258)	0.602	1.46 (0.482)	0.00	1.066(0.827 – 1.373)	0.623	4.22 (0.121)	52.6	Y

Chi_H_^2^ and P_H_: chi-squared and P values of the Q-test of heterogeneity; HNPCC: hereditary nonpolyposis colorectal cancer; sCRC: sporadic colorectal cancer; sCC: sporadic colonic cancer; PB: population-based study; HB: hospital-based study; PCR: polymerase chain reaction; RFLP: restriction fragment length polymorphism; SSCP: single-stranded conformation polymorphism; HPLC: high-performance liquid chromatography; P_E_: Egger’s test in each model; Y: yes; *: pooled OR was replaced with RD.

### Stratifying Analyses

**Figure 3:**
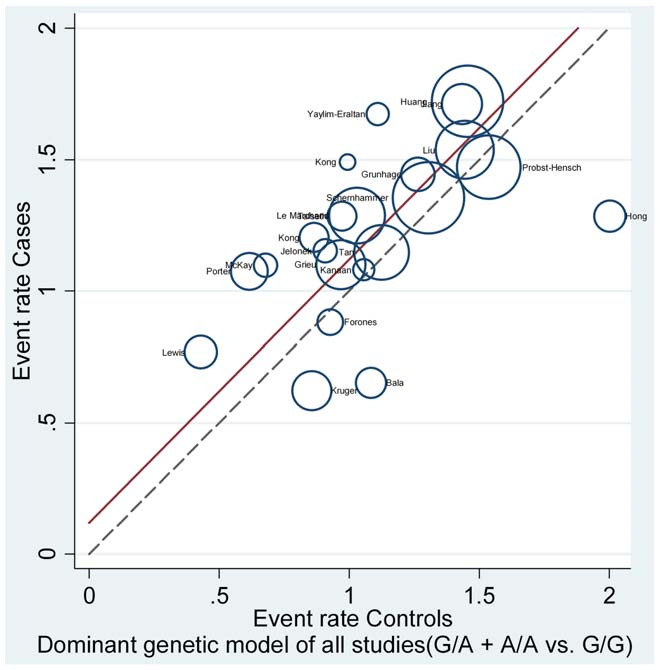
The L’Abbé plot [**14**] for the assessment of CRC risk in each group (G/A+A/A vs. G/G). Each circle represents individual trial sizes, and the circles are proportional to the study weights (participant number). The diagonal dotted line indicates that the CRC risk was equal in the two arms within the trials. The solid regression line represented a summary OR of 1.127 (G/A+A/A vs. G/G), which was estimated from the pooled results of all 22 studies.

We conducted subgroup analyses, and the results are listed in **Table 2**. Additionally, the L’Abbé plot was also used to assess the CRC risk in each group in all included studies (**Figure 3**).

Significant association of the *CCND1* G870A polymorphism with CRC risk was observed in many subgroup categories, including subsets of hospital-based studies (A/A vs. G/G: OR = 1.260, 95% CI = 1.072–1.482, P = 0.005; G/A vs. G/G: OR = 1.249, 95% CI = 1.082–1.442, P = 0.002; G/A+A/A vs. G/G: OR = 1.252, 95% CI = 1.093–1.433, P = 0.001), subsets of sCRC cases (G/A vs. G/G: OR = 1.204, 95% CI = 1.053–1.376, P = 0.007; G/A+A/A vs. G/G: OR = 1.188, 95% CI = 1.046–1.348, P = 0.008), subsets of Caucasian ethnicity (G/A vs. G/G: OR = 1.145, 95% CI = 1.004–1.306, P = 0.043; G/A+A/A vs. G/G: OR = 1.162, 95% CI = 1.026–1.316, P = 0.018), subsets of Duke’s stage C/D (A/A vs. G/G: OR = 1.275, 95% CI = 1.007–1.613, P = 0.043; G/A vs. G/G: OR = 1.365, 95% CI = 1.097–1.698, P = 0.005), subsets of the well/moderate degree of differentiation (G/A+A/A vs. G/G: OR = 1.337, 95% CI = 1.063–1.682, P = 0.013), male subjects (G/A vs. G/G: OR = 1.393, 95% CI = 1.073–1.809, P = 0.013; G/A+A/A vs. G/G: OR = 1.359, 95% CI = 1.080–1.710, P = 0.009), and subsets of the PCR-RFLP genotyping method (A/A vs. G/G: OR = 1.262, 95% CI = 1.126–1.415, P<0.001; G/A vs. G/G: OR = 1.190, 95% CI = 1.076–1.315, P = 0.001; G/A+A/A vs. G/G: OR = 1.216, 95% CI = 1.106–1.337, P<0.001). Specifically, the subgroup of Caucasian ethnicity was associated with 1.3- to 1.5-fold increased risk of sCRC without heterogeneity (A/A vs. G/G: OR = 1.511, 95% CI = 1.158–1.972, P = 0.002; G/A vs. G/G: OR = 1.307, 95% CI = 1.057–1.617, P = 0.014; G/A+A/A vs. G/G: OR = 1.369, 95% CI = 1.118–1.676, P = 0.002) (**Table 2**).

### Sensitivity Analyses

Sensitivity analyses was performed by omitting one study at a time. This procedure did not influence the pooled value, which supports the robustness of this current meta-analysis.

### Publication Bias Analysis

**Figure 4:**
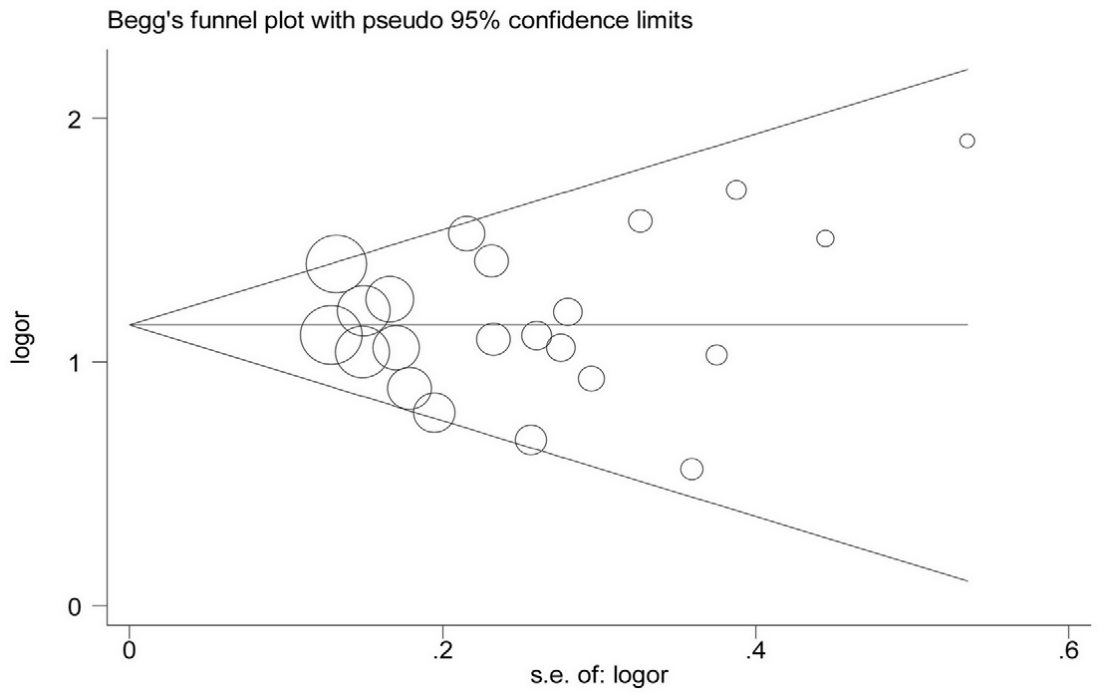
Begg’s funnel plot [**17**] (GA vs. GG) for the identification of publication bias in all studies.

The Begg’s funnel plot and the Egger’s test (A/A vs. G/G: P = 0.465; G/A vs. G/G: P = 0.731; G/A+A/A vs. G/G: P = 0.516; A/A vs. G/G+G/A: P = 0.399) showed no evidence of publication bias (**Figure 4**).

### Assessment of Cumulative Evidence

We applied the Venice criteria [19] to evaluate the overall evidence of an association between the *CCND1* G870A polymorphism and colorectal cancer susceptibility. The total sample size (6157 cases and 8198 controls) in our meta-analysis exceeded 1000. Therefore, we assigned the amount of evidence category an A grade. Next, we assessed the extent of replication. Our meta-analysis showed a significantly increased risk of colorectal cancer in the homozygote genetic model, the heterozygote genetic model and the dominant genetic model but not in the recessive model in any category. We observed minimal heterogeneity in the heterozygote genetic model and the dominant genetic model and moderate heterogeneity in the heterozygote genetic model. Therefore, we assigned a B grade for the extent of replication. Finally, there was no evidence of publication bias in our pooled data, and most of the included studies were well matched for race, ethnicity, gender and age. The summary ORs of each genetic model were greater than 1.15; therefore, bias could not have easily rendered the observed association. Nevertheless, most studies did not publish sufficient information about whether the G870A polymorphism was relevant to other polymorphisms or other candidate genes. Therefore, the Venice criterion of protection from bias was given a B grade. The overall grade of the Venice criteria for our data was “ABB”, which is consistent with moderate evidence demonstrating the linkage between the G870A polymorphism and colorectal cancer risk.

## Discussion

Cell cycle regulation plays an important role in the evolution of cancer by influencing cell proliferation, differentiation and apoptosis [43]. It has been demonstrated in all eukaryotic organisms that the transition from the G1 phase to the S phase of the cell cycle is controlled by sequential activation of cyclin/cyclin-dependent kinase (Cdk) complexes [44]. The cyclin D1 locus (also called *CCND1* or PRAD1, located on 11q13) consists of five exons and four introns and encodes cyclin D, a key regulatory protein promoting the transition through the restriction point in the G1 phase [45]. Over 250 single nucleotide polymorphisms (SNP) spanning *CCND1* have been identified and cataloged in public SNP databases (dbSNP: www.ncbi.nlm.nih.gov/SNP/; HapMap: www.hapmap.org). Of the polymorphisms identified, the common adenine-to-guanine (A/G) substitution at nucleotide 870 in the conserved splice donor region of exon 4 has received the most investigation [6]. Normally, the G870 allele creates an optimal splice donor site and results in a well-described transcript for cyclin D1, termed cyclin D1a; however, the *CCND1* G870A polymorphism at the boundary of exon 4 and intron 4 affects alternative splicing and results in an variant transcript for cyclin D1, termed cyclin D1b, which lacks exon 5 [6], [46], [47]. Therefore, cyclin D1b is homologous to cyclin D1a but lacks two regulatory motifs, the point estimation by sequential testing (PEST) domain and the threonine 286 phosphorylation site for glycogen synthase kinase 3ß, both of which are crucial in preventing the overexpression of cyclin D1 [6], [46], [47]. Excessive cyclin D1 activates CDK4/cyclin D1 complexes and initiates the phosphorylation of RB, which disrupts RB-mediated transcriptional repression of E2F and facilitates cell cycle progression [48], [49].

The current meta-analysis and systematic review summarizes the results from 22 case-control studies on the association of the *CCND1* G870A polymorphism with CRC risk. A total of 6157 cases and 8198 controls were included. Based on the Venice criteria, the results indicated that the G/A or A/A genotype of *CCND1* SNP rs603965 was significantly associated with an increased risk of CRC. Additionally, we found no significant risk of CRC associated with the *CCND1* G870A polymorphism for the recessive model in any category, indirectly suggesting the linkage of the A-allele and increased CRC risk.

In the stratified analyses, the results showed that the association between the *CCND1* G870A polymorphism and CRC risk remained significant in Caucasians and sCRC but not in Asians or HNPCC, which supports the hypothesis that genetic backgrounds and the environment in which patients live in might play important roles in the development of CRC [5]. Meanwhile, the finding that no association between the *CCND1* genotype and CRC risk was observed in the comparison model of either the colon subgroup or the rectum subgroup was in contrast with the results from another meta-analysis investigating digestive tract cancers and the risk associated with the *CCND1* G870A polymorphism [50]. We also found a significant association between G870A and CRC risk in a subset of hospital-based studies but not in the population-based studies. The lack of proper matching of controls among the studies might influence the consistency in our current results.

Meta-analysis is an important tool for revealing trends that might not be apparent in a single study. The pooling of independent but similar studies increases precision and therefore increases the confidence level of the findings. The current meta-analysis has some advantages. First, the number of total cases and controls was substantial, which significantly increased the statistical power of the analysis. Second, no publication biases were detected, which indicates that the entire pooled result may be unbiased.

Despite these advantages, some limitations in the current meta-analysis should be acknowledged. First, the controls were not uniformly defined. Although most of the patients in the control groups were selected from healthy populations, some might have had a benign disease. Therefore, there was a lack of proper matching, and the results are based on unadjusted estimates. The current meta-analysis is unable to solve problems with confounding factors that could be inherent in the included studies. Inadequate control of the confounders might bias the results either toward exaggeration or underestimation of risk estimates. Second, stratifying analyses were based on a relatively small number of studies from which detailed individual data were available; therefore, some of the subgroup analyses were difficult to perform. Third, although there is no indication of major publication bias in the formal evaluation used, potential publication bias is impossible to completely exclude because small studies with null results tend to not be published. Finally and mostly importantly, whether the *CCND1* G870A polymorphism is independently predictive of cancer risk remains controversial [6], [51]. Thus, it should be noted that whether the A allele is a specific causal variant has yet to be determined. Some functional studies have demonstrated that the G allele can also produce transcript b (cyclin D1b), and the A allele can also produce transcript a (cyclin D1a)[22], [51], [52]; these results suggest that the A allele is not universally required for transcript b (cyclin D1b) production. Furthermore, one study demonstrated that the G870A and G1722C polymorphisms of cyclin D1 were in linkage disequilibrium in carcinomas of the head and neck [52]. Another study demonstrated that there was a synergistic effect between *CCND1* G870A and caspase−8 6 n del/ins on CRC [40]. Therefore, it is possible that G870A is in linkage disequilibrium with another functional variant that modulates cancer risk. Additionally, there is no genome-wide association study (GWAS) identifying the susceptibility loci of *CCND1* for colorectal cancer, although one group recently published a GWAS in which *CCND1* was strongly suggestive in melanoma carcinogenesis [53]. Hence, large, prospective, population-based clinical trials and genome-wide association studies are required to validate the association of the *CCND1* G870A polymorphism with CRC risk.

In conclusion, the current meta-analysis and systematic review demonstrated that the *CCND1* G870A polymorphism is associated with CRC susceptibility, especially among patients of Caucasian ethnicity. The current results may prompt further investigation of diagnostic approaches and prevention strategies to combat CRC.
